# Endovascular coil embolization of inferior mesenteric artery to ileal-conduit fistula: a case report

**DOI:** 10.1186/s12894-022-00961-5

**Published:** 2022-01-31

**Authors:** Mustafa A. Altaha, Massimo Tarulli, Jaspreet Bajwa, Sebastian Mafeld, Arash Jaberi

**Affiliations:** 1 Division of Interventional Radiology, University Health Network, Toronto General Hospital, Toronto, ON Canada; 2 Diagnostic and Interventional Radiology Resident, Joint Department of Medical Imaging, University of Toronto, 1 Dunsmore Gdns., Toronto, ON M3H 3M1 Canada

**Keywords:** Hematuria, Uretero-arterial fistula, Ileal-conduit, Ureteral stent, Case report

## Abstract

**Background:**

Uretero-arterial fistulas (UAFs) are uncommon and pose a diagnostic dilemma, making them life threatening if not recognized and treated expediently. UAFs to small arteries such as a branch of the inferior mesenteric artery (IMA) are very uncommon and present a further diagnostic and treatment challenge. There should be a high index of suspicion for UAFs when intervening on patients with history of treated pelvic cancers and long-standing ureteric stents experiencing hematuria not attributable to another cause.

**Case presentation:**

We present a case of a fistula formed between a distal branch of the IMA—superior rectal artery—and an ileal-conduit in a patient with a long-standing reverse nephroureterostomy (Hobbs) catheter presenting with abdominal pain and hematuria through the conduit. During a tube exchange, contrast injection demonstrated a fistula with the superior rectal artery, multiple ileal intraluminal blood clots, and active extravasation. The patient became tachycardic and hypotensive, actively bleeding through the ileal-conduit, prompting a massive transfusion protocol. Successful endovascular coiling of the superior rectal artery was performed with resolution of active extravasation and stabilization of the patient. The patient recovered and was discharged in stable condition 10 days later.

**Conclusions:**

Although UAFs are uncommon, our case demonstrated key predisposing risk factors to fistula development; pelvic cancer surgery, pelvic radiation, and a prolonged ureteric stent through the ileal-conduit. Typically, UAFs arise from communication with the iliac arterial system, however in this instance we have demonstrated that fistulization to other arterial vessels is also possible. Endovascular management has become the preferred method of therapy, typically involving the placement of covered stents when involving the iliac arterial system. In this instance stent grafting was not possible due to the small caliber vessel and therefore had to be embolized.

## Background

Uretero-arterial fistula (UAF) is relatively rare with only 150–162 cases described in literature. The incidence of UAF is increasing secondary to improved pelvic cancer treatment and survival [1–3]. Reported UAFs predominantly communicate with the iliac arterial system. Roderick et al., described 139 UAFs in a literature review, with 132 (95%) communicated with the iliac artery or vascular graft material in the aortoiliac trajectory; remaining 7 cases communicated with the aorta [4].

The diagnosis of UAF should be considered in patients presenting with hematuria not attributable to a specific cause, with or without pain, fever, and hydronephrosis [1, 2, 5].

Since the early 2000s, endovascular treatment has become the preferred management method, reserving open surgery to cases of enteric contamination, abscess formation, and infected stents/failed endovascular treatment [2, 6–9].

Here we present a unique case of fistulous communication between an ileal-conduit and the superior rectal artery (distal branch of the IMA), with resuscitation and prompt endovascular treatment. A review of literature is provided.

## Case presentation

### Background and diagnostic work-up

**Fig. 1:**
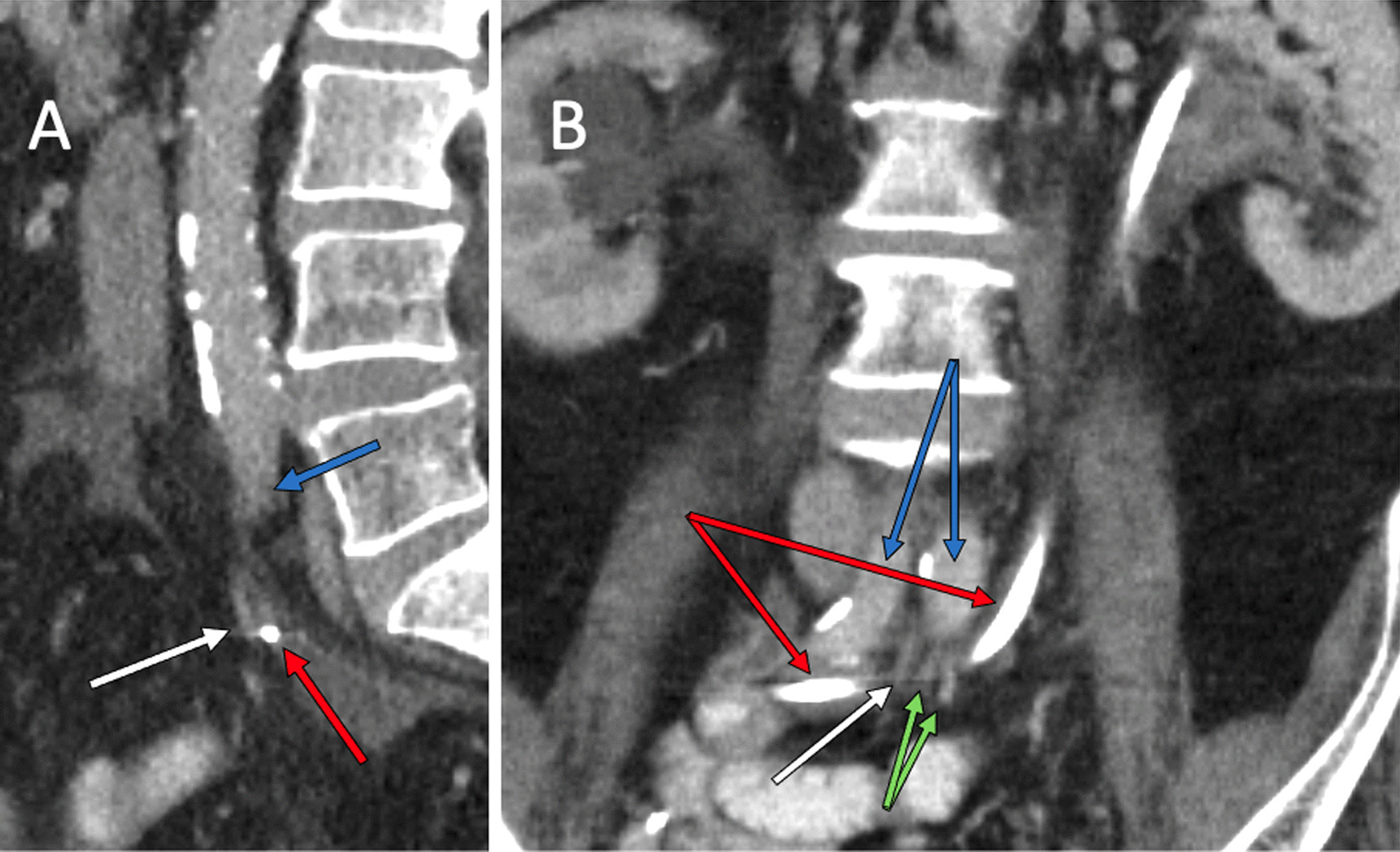
CTA of the abdomen and pelvis performed two months prior to the endovascular intervention, demonstrated intimate posterior relationship (**A**) of the reverse Hobbs catheter (red arrow) to three distal IMA branches (white arrow for culprit vessel and green arrows for remaining branches (**A**, **B**)), and close anterior position of Hobbs catheter to common iliac vessels (blue arrows). Note is made of bilateral hydronephrosis and left ureteropelvic blood clots

A 65-year-old male with a history of recurrent stage 3A bladder cancer following trimodal therapy and subsequent disease recurrence, a radical cystoprostatectomy and ileal-conduit formation was performed. The patient developed early strictures at the distal left ureteral anastomoses with hydronephrosis. The stricture was treated with balloon ureteroplasty and insertion of a reverse nephroureterostomy (Hobbs) catheter. The patient later developed right sided hydronephrosis, with nephrostomy placed after failed attempt at reverse Hobbs insertion. Around the same time, the patient was complaining of significant abdominal and flank pain for which no obvious cause was identified. This was in the context of persistently low hemoglobin (< 100) for approximately 12 months. He was started on low dose apixaban after two recent episodes of pulmonary embolism, and later developed duodenal ulcers. His creatinine had been increasing steadily to > 200. He also developed septicemia with elevated neutrophils. A month later, the patient presented to the Emergency Department with new presyncope and 300 ml of dark red blood in the ileal-conduit ostomy appliance. The left reverse Hobbs was exchanged as it had migrated into the distal left ureter. Four days later, it had migrated again, thought to be due to multiple clots dilating the left collecting system, with ongoing blood product through the ileal-conduit raising suspicion of a UAF. Of note, the right nephrostomy bag was clear. Upon consultation with interventional radiology, preparation was made to provide endovascular treatment if active hemorrhage was elucidated. A prior staging CT had demonstrated the reverse Hobbs catheter traversing closely anterior to the common iliac arteries bilaterally, as well as intimately posterior to the distal IMA branches (Fig. 1), with left hydronephrosis (likely secondary to blood products in the collecting system).

### Intervention

**Fig. 2:**
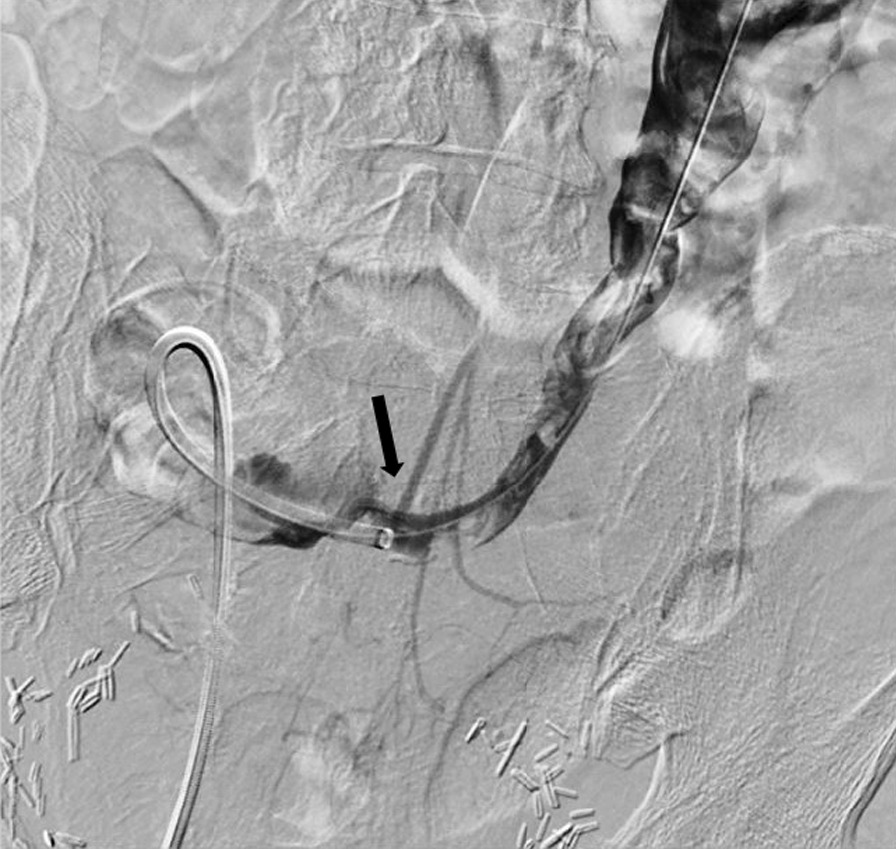
Digital subtraction ureterogram with contrast injected as the sheath was retracted demonstrating a fistula between the distal ureter and an IMA branch (black arrow). Also seen, multiple filling defects within the left renal pelvis and left dilated proximal ureter, consistent with blood clots

The patient arrived in our department vitally stable and informed consent was obtained. A right nephrostomy and left reverse Hobbs were in-situ. The urostomy appliance contained sanguineous fluid. The existing left reverse Hobbs had retracted compared to previous. Injected contrast opacified the ileal-conduit, but not the left ureter and renal pelvis suggesting possible distal blockage (likely by blood products). The tube was rewired and removed over a super stiff Amplatz wire and mixed blood products were seen from the ostomy, suggesting active hemorrhage. At this point, the patient remained vitally stable. A 10Fr, 40 cm Flexor Check-Flo II Introducer sheath was quickly advanced over the wire to tamponade and perform a nephroureterogram while retracting the sheath to interrogate the ureter. Multiple filling defects were seen within the renal pelvis and dilated proximal ureter, consistent with blood clots (Fig. 2). Within the distal ureter, there was faint filling of a tubular structure intersecting with the ureter, likely a vessel branch. Digitally subtracted ureterogram was performed demonstrating a fistula between the distal ureter and an IMA branch (Fig. 2).

**Fig. 3:**
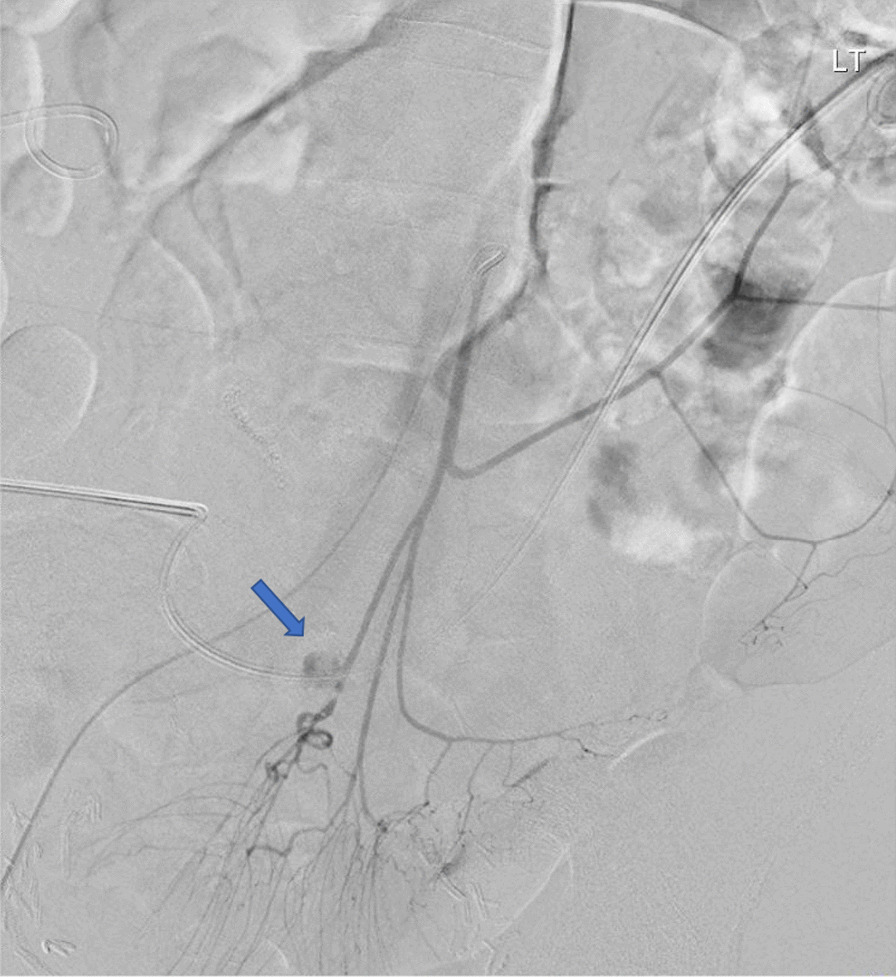
Digitally subtracted angiogram demonstrates active extravasation of the superior rectal artery into the ileal-conduit (blue arrow)

The patient began to bleed more briskly and became unstable (tachycardia to the 160 s bpm), prompting a Code Omega (massive transfusion protocol) to be called. Colloid resuscitation was initiated at maximal rate. A 10 Fr reverse Hobbs was inserted to tamponade. Ultrasound guidance and Seldinger technique was used for retrograde right CFA access. A 5-Fr SOS with Bentson wire were used to engage the IMA. Angiogram demonstrated active extravasation of the superior rectal artery into the ileal-conduit (Fig. 3).

**Fig. 4:**
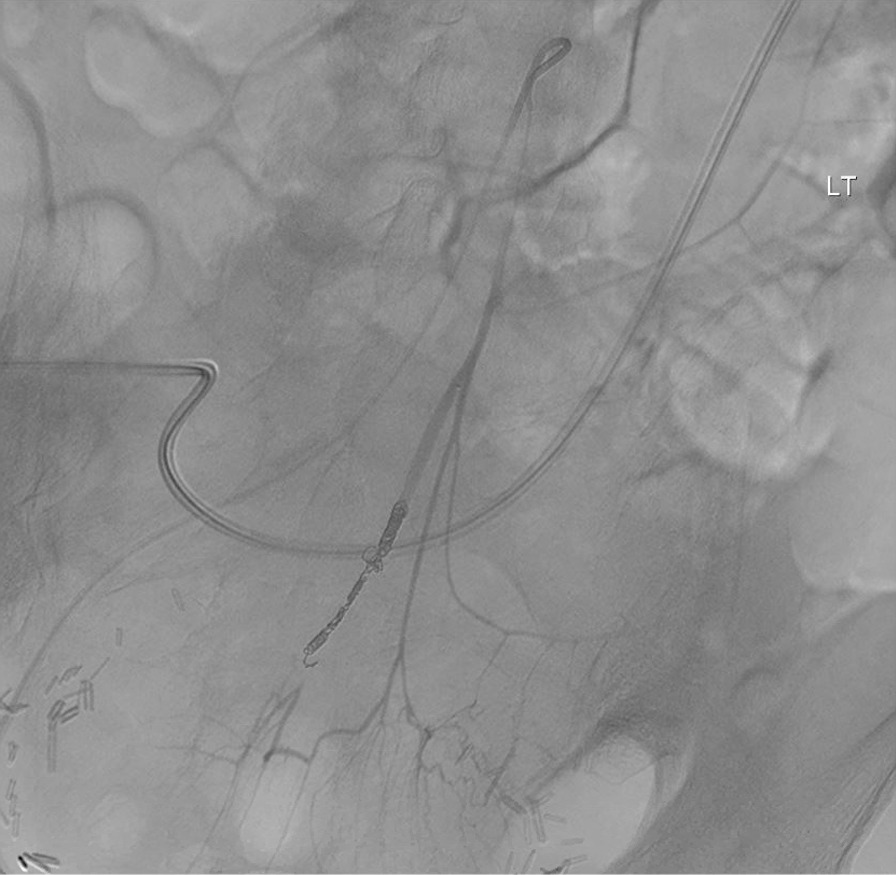
Digitally subtracted angiogram of the IMA demonstrated cessation of flow through the proximal superior rectal artery in the region of the intersection between the artery and ureter with retained perfusion of the rectosigmoid region and resolution of active extravasation

A 2.4 Fr Progreat microcatheter and 0.018″ Gold-tip glide wire were advanced beyond the extravasation into the mesenteric portion of the superior rectal artery, and coil embolization was performed across the intersection between the IMA and ureter with eight 5-2 mm Tornado Microcoils (0.018in). Subsequent angiography demonstrated exclusion of the segment of extravasation, preserving distal perfusion of the rectosigmoid region (Fig. 4). The patient was resuscitated with three units of blood and stabilized relatively quickly. The Hobbs catheter was retracted over the wire, and angiogram was repeated to confirm resolution of active extravasation. The 10-Fr reverse Hobbs catheter was reinserted. The patient was stable enough to be transferred to the floor.

### Discharge and follow-up

The patient recovered in the hospital for another 10 days and was discharged.

**Fig. 5:**
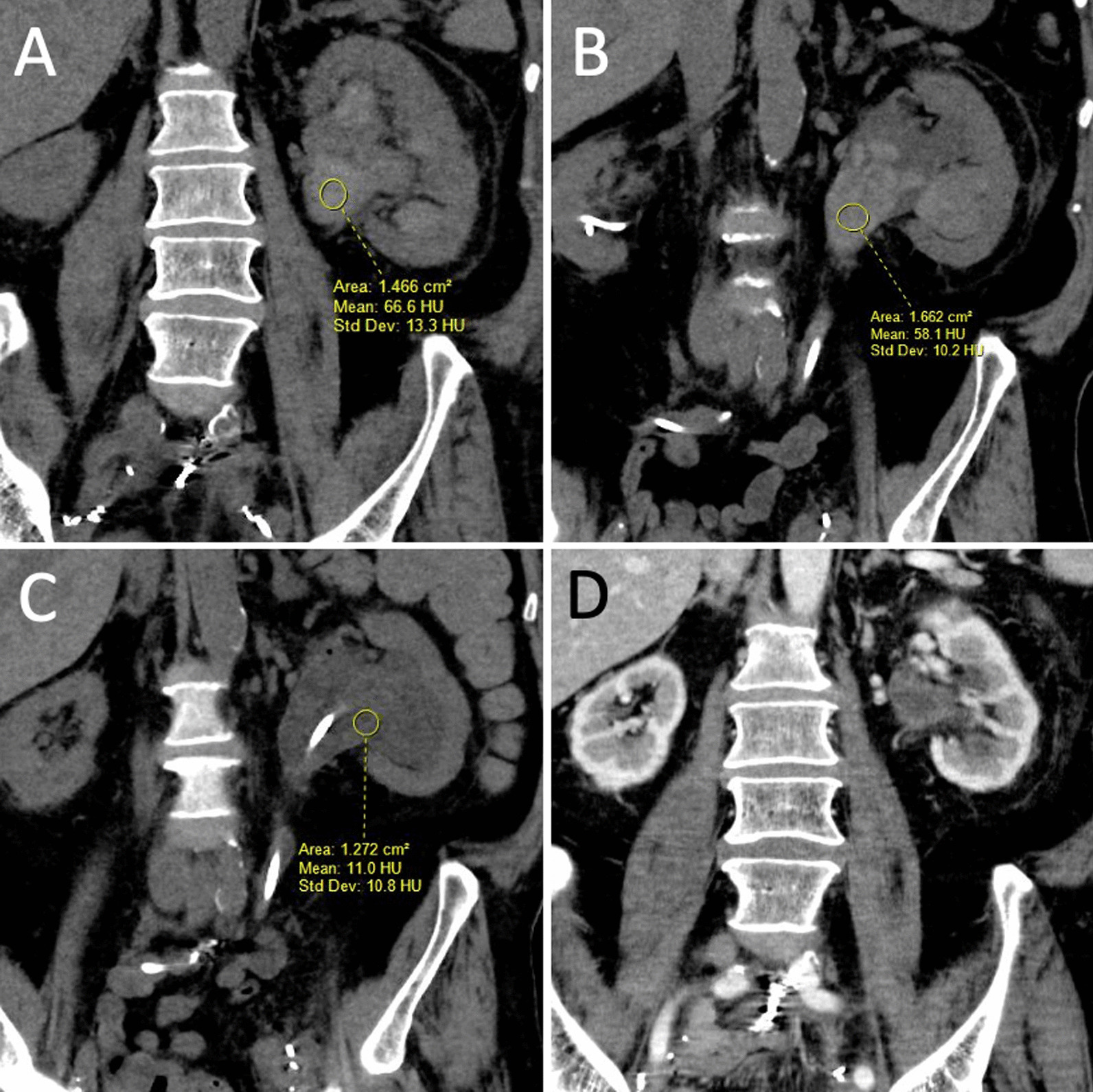
CT of the abdomen and pelvis at different time points, demonstrate large blood clots in the left pelviureteric system with significant left hydronephrosis not significantly different between two consecutive CT scans, **A** from 11 days prior to- and **B** from two days post- embolization. **C** Interval resolution of pelviuretric dense blood clots 17 days post embolization, but persistent significant hydronephrosis. **D** Interval resolution of hydronephrosis 45 days post embolization; no evidence of recurrence of dense blood clots

CT imaging two days post embolization demonstrated persistent left hydronephrosis, and blood clots within left renal pelvis and proximal ureter, similar to 11 days prior to embolization, which was not unexpected, (Fig. 5A, B). Follow up imaging a month later demonstrated resolution of blood clots within the left urinary system, with persistent left hydronephrosis, (Fig. 5C). The left reverse Hobbs was removed, and left nephrostomy inserted without recurrent bleeding, (Fig. 5D).

## Discussion and conclusion

To our knowledge, we report the first case of a fistulous connection between an ileal-conduit and inferior mesenteric artery branch “superior rectal artery” in a patient who presented with classical clinical features of UAF. The patient had the three most common predisposing risk factors for the development of UAF, namely, prior oncologic pelvic surgery, pelvic radiation, and prolonged ureteral stent placement [3, 5] Pelvic surgery predisposes to distal ureteric strictures [10]. Radiation induces inflammation and fibrosis causing impaired wall compliance leading to increased wall tension [11, 12]. Pelvic radiation in isolation, however, causes predominantly non-vascular urologic fistulas, which are more likely to occur to the bowel or to vagina/uterus [12]. Prolonged ureteral stent placement (or catheterization) was likely a contributing factor to UAF in our patient. Ureteric stents are commonly employed when distal ureteric strictures ensue, which is a relatively common late complication. Causes for this include fibrosis at the distal ureteric junction resulting from rejection or ischemia, such that due to operative technique and/or immunosuppression [10, 13]. Constant arterial pulsations against a traversing firm intra-urinary stent has been suggested to lead to pressure necrosis, with subsequent fistula formation [11]. Furthermore, ureteral reconstruction has been shown to compromise healing around areas of pressure necrosis, which further predispose to fistula formation [14, 15]. Roderick et al. therefore, recommended against placing the uretero-ileal-conduit anastomosis near traversing vessels to limit vascular scaring and UAF formation [4].

Our literature review demonstrated 42 cases of UAF in patients with ileal-conduits. The vast majority communicated with the iliac arteries (97%). It was more likely to communicate with the common iliac artery (72%) than with branch iliac arteries (internal iliac 16% and external iliac 12%); it was however, not specified to which iliac artery in 13 patients. Iliac artery fistula happened to be right sided in 82% of described UAFs, compared to the left as in our case. The fistulous tract communicated with the conduit in about one-third of cases (arterial ileal-conduit fistula) like in our case, and with the ureter in two-thirds (typical UAF); it was not specified in about 60%, however. The first arterial ileal-conduit fistula was reported in 1971 [16, 17]. Twelve of the 42 cases that we identified of UAF in patients with ileal-conduit were reported between 1971 and 1990 [11]. We did not find any reported case of arterial-ileal-conduit fistula that involved the inferior mesenteric artery or its branches in the English literature. Moreover, in a systematic review, Jose et al. reported a total of 94 cases of UAFs, none of which involved the inferior mesenteric artery [2]. UAFs predominantly develop at the crossing common iliac or branch iliac arteries [9, 18]. UAFs involving the aorta, renal artery, bilateral iliac arteries (double fistulae), or other pelvic arteries are exceedingly rare but can occur [2, 18, 19].

UAF remains uncommon with the total number of cases in literature was estimated in 2018 to be 150–162 cases [1, 2]. These numbers are likely underestimated. For instance, Rafael et al. and Safwan et al. each reported their separate single center experience with UAF to be 21 cases and 27 cases, between 1996–2010, and 2011–2020, respectively [6, 8].

We have presented a case of successful endovascular UAF embolization by coiling of the segment of superior rectal artery contacting the branch IMA-ileal-conduit fistula. We placed multiple small coils (8 micro-coils 5 × 2 mm) rather than a longer spiral (e.g. 5 × 15 or 5 × 20 mm) since smaller coils are easier to control in terms of precise deployment. This allows for preserving collaterals. We avoided the use of high viscosity liquid embolic agents because they pose higher risk of non-target embolization, including distal embolization with bowel ischemia. An alternative method of endovascular treatment of UAF is with endovascular covered stent placement, which has shown good results especially in patients with arterial-ileal-conduit fistula, however, is limited to larger vessels [2, 6–9]. Following endovascular UAF embolization, our patient demonstrated resolution of hydronephrosis and hematuria and experienced a slow but steady clinical improvement within the 45-day follow up period, including improvement in hemoglobin, creatinine, and white blood cell count.

In summary, although UAFs are uncommon, our case demonstrated key predisposing risk factors to fistula development; pelvic cancer surgery, pelvic radiation, and a prolonged ureteric stent through the ileal-conduit. Typically, UAFs arise from communication with the iliac arterial system, however in this case we have demonstrated that fistulization to other arterial vessels is also possible. Endovascular management has become the preferred method of therapy, typically involving the placement of covered stents when involving the iliac arterial system. In this instance, stent grafting was not possible due to the small caliber vessel and therefore had to be embolized.

## Data Availability

The literature review data analyzed during the current study are available from the corresponding author on reasonable request.

## Abbreviations

IMA: Inferior mesenteric artery
UAF: Uretero-arterial fistula
Hobbs: Nephroureterostomy
